# Mining synergistic genes for nutrient utilization and disease resistance in maize based on co-expression network and consensus QTLs

**DOI:** 10.3389/fpls.2022.1013598

**Published:** 2022-10-28

**Authors:** Bowen Luo, Jiaqian Li, Binyang Li, Haiying Zhang, Ting Yu, Guidi Zhang, Shuhao Zhang, Javed Hussain Sahito, Xiao Zhang, Dan Liu, Ling Wu, Duojiang Gao, Shiqiang Gao, Shibin Gao

**Affiliations:** ^1^ State Key Laboratory of Crop Gene Exploration and Utilization in Southwest China, Chengdu, Sichuan, China; ^2^ Maize Research Institute, Sichuan Agricultural University, Chengdu, Sichuan, China; ^3^ Key laboratory of Biology and Genetic Improvement of Maize in Southwest Region, Ministry of Agriculture, Chengdu, Sichuan, China; ^4^ Key Laboratory of Wheat and Maize Crops Science, College of Agronomy, Henan Agricultural University, Zhengzhou, China

**Keywords:** Nutrition utilization, disease resistance, synergistic genes, co-expression network, cQTLs, maize

## Abstract

Nutrient restrictions and large-scale emergence of diseases are threatening the maize production. Recent findings demonstrated that there is a certain synergistic interaction between nutrition and diseases pathways in model plants, however there are few studies on the synergistic genes of nutrients and diseases in maize. Thus, the transcriptome data of nitrogen (N) and phosphorus (P) nutrients and diseases treatments in maize, rice, wheat and Arabidopsis thaliana were collected in this study, and four and 22 weighted co-expression modules were obtained by using Weighted Gene Co-expression Network Analysis (WGCNA) in leaf and root tissues, respectively. With a total of 5252 genes, MFUZZ cluster analysis screened 26 clusters with the same expression trend under nutrition and disease treatments. In the meantime, 1427 genes and 22 specific consensus quantitative trait loci (scQTLs) loci were identified by meta-QTL analysis of nitrogen and phosphorus nutrition and disease stress in maize. Combined with the results of cluster analysis and scQTLs, a total of 195 consistent genes were screened, of which six genes were shown to synergistically respond to nutrition and disease both in roots and leaves. Moreover, the six candidate genes were found in scQTLs associated with gray leaf spot (GLS) and corn leaf blight (CLB). In addition, subcellular localization and bioinformatics analysis of the six candidate genes revealed that they were primarily expressed in endoplasmic reticulum, mitochondria, nucleus and plasma membrane, and were involved in defense and stress, MeJA and abscisic acid response pathways. The fluorescence quantitative PCR confirmed their responsiveness to nitrogen and phosphorus nutrition as well as GLS treatments. Taken together, findings of this study indicated that the nutrition and disease have a significant synergistic response in maize.

## Introduction

N and P are essential macronutrients for plant growth and development. In most natural systems, N and P are the predominant rate-limiting nutrients and the major constituents of agrochemical fertilizers (Guignard et al., 2017). Adequate nutrients are the prerequisite to ensure the optimal growth and development as well as stable and high yield of crops. However, an excess or a deficiency of nutritional elements will limit crop development and hasten the emergence of plant diseases.

N is an essential element for plant growth and is closely linked to the incidence of plant diseases. The sensitivity of tomato to *Fusarium oxysporum* and grape to *Botrytis cinerea* rose under the N deficiency conditions, but a high concentration of N would increase the sensitivity of tomato to powdery mildew and bacterial spot pathogen (Hoffland et al., 1999; Hoffland et al., 2000). The application of N fertilizer exacerbated the occurrence of southern leaf wilt and stem rot disease in maize (Manching et al., 2014; Abiodun et al., 2015). Moreover, Talbot et al. (1997) found that nitrogen starvation induced the expression of a large number of genes in rice, especially during disease outbreaks. *Fnr1* is a gene related to N metabolism pathway which can affect the growth, development and toxic expression of *Fusarium oxysporum* (Divon et al., 2006). Furthermore, two genes *NPR1* and *NPR2* (for N pathogenicity regulation), were identified, and both are required for the utilization of N sources and pathogenicity (Lau and Hamer, 1996; Talbot et al., 1997). *GS2* and nitrate reductase genes involved in N assimilation were down-regulated during plant-pathogen interaction, whereas *GS1* and *GDH* genes involved in N retransfer were up-regulated (Pageau et al., 2006; Tavernier et al., 2007). N can also regulate biotic defense *via* amino acid metabolism and hormone production, influencing the expression of downstream defense-related gene *via* transcriptional regulation and nitric oxide (NO) production, indicating a direct relationship with N (reviewed by Sun et al., 2020).

P is known to promote the growth of the root system and enhance the absorption of nutrients, hence minimizing the impact of root diseases on plants (Brennan, 1995). However, sufficient phosphate fertilizer has been reported to greatly increase the incidence area of black spot, decrease the content of phenol, and weaken the resistance of plant to bacterial blight (Sharma and Kolte, 1994). *PHR1*, as the central regulator of the response to low P stress, directly regulates the expression of immune-related genes (Castrillo et al., 2017). The salicylic acid (SA) response defense genes were found to be generally up-regulated when *PHR1* was mutated (Lebeis et al., 2015). This finding is consistent with the increased resistance phenotype of *phr1* and *phr1phl1* mutants to *Pseudomonas syringae* DC3000 infection (Castrillo et al., 2017), and the *PHR1* was found to negatively regulate immune defense response under low P conditions. Other studies have found significant effects of P transporters and plant hormones on the signal fusion between cellular P and immune response, in addition to external soil P availability, plant intracellular P and P starvation signaling mechanisms (reviewed by Chan et al., 2021). In summary, while recent studies have revealed insights into the interplay between nutrition and immune signaling pathways, how these responses are integrated remains largely unknown (reviewed by Gong et al., 2020).

According to the most recent review studies (Zhang et al., 2019; Chu et al., 2021), the research of nutrient utilization and disease resistance in plants has made great progress. A number of critical genes with substantial breeding value have been cloned, and the signal networks have been studied from signal reception and transduction to functional proteins. However, these studies only focused on nutrient utilization or disease resistance alone, leaving the cross-study of nutrient and disease resistance signals largely unexplored. On the other hand, previous studies on nutrition and diseases have only focused on the effects of individual functional elements (such as DNA, mRNA and protein) on life activities at the molecular level. Although this method is of great significance for revealing the genetic mechanism of specific traits, traditional biological research alone is insufficient for analyzing the complex quantitative traits such as the nutrition- and disease-associated traits. As a powerful method to study QTL, genetic mapping has been widely used to locate the loci controlling the target quantitative traits to very small intervals or directly to a single gene (Yan et al., 2011). Several studies have been reported on large number of QTL in maize, making it a suitable source for both nutrition- and disease-related QTL (Yan et al., 2011; Zhang et al., 2014; Luo et al., 2015). Furthermore, the development of an effective data mining method, WGCNA, it enables the establishment of co-expression gene modules with high biological significance based on specific trait-related gene screening and classification (Langfelder and Horvath, 2008; Schaefer et al., 2018; Guo et al., 2020; Ma et al., 2021). As a result, by integrating the results of WGCNA and QTLs, the target genes can be accurately screened out.

Maize is not only the largest food crop in China, but also an important industrial raw resource around the world. Nutrient scarcity and widespread prevalence of associated diseases have emerged as major problems limiting agricultural productivity. To secure the crop yield, enormous applications of N and P fertilizer, as well as chemical control of diseases across a large area, have seriously damaged the environment and increased production costs (Ab Rahman et al., 2018; Dai et al., 2020). Therefore, improving the utilization efficiency of fertilizer and disease resistance becomes urgent. Moreover, exploring the relationship between nutrition and diseases may someday aid in field disease control by synchronizing fertilizer use. GLS is a devastating foliar disease caused by Cercospora (*Cercospora zeae-maydis* and *C. zeina*) infection (Tehon and Daniels, 1925). Due to the high incidence in southwest China where the geographical and climatic conditions are suitable for growth and reproduction of Cercospora (Shi et al., 2014; Sun et al., 2021), GLS is posing a huge threat to maize production in the major maize producing area in China. Therefore, the current work intended to dig out synergistic genes of both nutrition and diseases by combining the WGCNA and meta-QTL analysis. In particular, we took the GLS as an example to verify the entangled signal pathways between nutrition and diseases of maize, which might be possible for us to control crop GLS by adjusting the fertilizer input.

## Materials and methods

### Plant materials and N, P and GLS treatments

The materials B73 and Mo17 from Maize Research Institute of Sichuan Agricultural University were used in this experiment. After drying the seeds in an oven at 37°C for 12h, they were washed three times with deionized water and disinfected for 40 minutes with 30% hydrogen peroxide. Then the seeds were steeped overnight in a saturated calcium sulfate solution and placed in a damp vessel during the daytime until the radicle developed. The seeds were transplanted into turface-MVP, a non-nutrient cultivation medium. Three seedlings of each genotype were planted at three leaves stage in a pot with a height of 19 cm and an inner diameter of 22 cm. Three days after transplanting, the complete nutritional solution was added. When the seedlings reached the six-leaf stage, stress treatments were carried out, including normal nutrition treatment (CK), low N stress (LN), low P stress (LP), low N plus GLS (LN/GLS), low P plus GLS treatment (LP/GLS) and GLS. Nutrition treatments were carried out in accordance with the nutrition formula described in **Supplementary Tables 1 and 2**. The following are treatments for GLS. *Cercospora zeae-maydis* was cultured in a 26°C incubator in full darkness using maize leaf powder plus CaCO_3_ agar (maize leaf powder 15g/L, CaCO_3_ 1.5g/L, Agar 15g/L, chloromycetin 0.0625g/L). After reaching the maximal sporulation concentration, the spores and hyphae were washed with 2% tweens water to form a spore suspension with a concentration of 3.2 x 10^10^ ~ 4.8 x 10^10^. The leaves were then surface-wound with an acupuncture needle. Fresh-keeping film was used to keep fungal blocks moistened on local maize leaves. The 10 ml spore suspension was sprinkled evenly per plant with a spray syringe, then a two-day dark treatment and a five-day moisturizing treatment were applied at the same time. Samples were taken 5 days, 10 days, 15 days and 20 days following nutrition and disease treatments, respectively.

### Transcriptome data analysis

The raw transcriptome data of maize, rice, wheat and Arabidopsis thaliana under LP, LN and disease treatments were downloaded from the SRA database (https://www.ncbi.nlm.nih.gov/sra/). FastQC (https://www.bioinformatics.babraham.ac.uk/projects/fastqc/) was used for quality control of the original data, and Trim Galore was used to remove the adapter sequences and low-quality reads (–quality 30 –phred33 –length 13 –stringency 5) (Martin, 2011). HISAT2 was used to map transcriptome sequencing data to the reference genome after downloading the reference genome of the corresponding species from Ensembl (https://plants.ensembl.org/index.html) (Kim et al., 2015). StringTie was then used to calculate the transcripts per million (TPM) (Pertea et al., 2015).

### Homology and collinearity analysis among species

The blast libraries of wheat, rice and Arabidopsis thaliana were constructed using protein sequences. The protein sequences of wheat, rice, and Arabidopsis thaliana were compared with the whole genome protein sequences of maize by using blast tool. From the blast results, the genes with the highest bit score were chosen. The TPM expression matrix constructed by maize and maize homologous genes was then used for WGCNA. MCscan software was used to analyze collinearity between maize and wheat, rice, and Arabidopsis thaliana (Tang et al., 2008).

### WGCNA and MFUZZ clustering

The previously constructed TPM matrix was separated into root and leaf tissues. For the highest data reliability, genes with a maximum expression of less than five were filtered out prior to the WGCNA analysis. For a detailed analysis process and parameters of WGCNA refer to previous studies (Langfelder and Horvath, 2008; Ma et al., 2021). Finally, the genes from the co-expression network modules obtained by WGCNA were imported into MFUZZ packets (Kumar and Futschik, 2007) for clustering. Clusters with obvious down- or up-regulation during nutrition and disease treatments were identified as a candidate cluster for further investigation.

### QTL meta-analysis

The QTL information of maize under LP, LN, disease and normal conditions was collected from the previous studies, and the QTL mapping results with incomplete information were discarded. The original maps that corresponded to QTLs were compared to the IBM2 2008 Neighbors reference map to ensure that each marker on the original maps was present on the reference map. The QTLs were then mapped to the IBM2 2008 Neighbors reference map (genes placed by recombinational and physical data, https://www.maizegdb.org/data_center/map) through BioMercator (v4.2) software. The Gauss theorem provides the best QTL models based on the maximum likelihood function ratio (Arcade et al., 2004). To acquire scQTLs related to both nutrition and disease treatments, cQTLs detected under both control and treated conditions were excluded, as were cQTLs that are not simultaneously associated with nutrition and disease related traits. Finally, the genes located in the scQTL regions were extracted.

### qRT-PCR analysis

The TRIzol Kit was used to extract total RNA (Invitrogen, Waltham, MA, USA). The mRNA was then reverse-transcribed using the PrimeScript™ RT Reagent Kit with gDNA Eraser (Perfect Real-Time) (TAKARA, Dalian, China) following the manufacturer’s instructions. The quantitative PCR primers for candidate genes are listed in (**Supplementary Table 3**) and were designed by Beacon Designer software (version 7.0; Premier Biosoft International, Palo Alto, CA, USA). The optimal Tm value and amplification efficiency were screened by Jena qTOWER3 G (Analytik Jena, Jena, Germany). Real-time quantitative PCR reactions were carried out on Roche Cobas Z480 (Roche Molecular Diagnostics, Pleasanton, CA). To normalize expression, the internal control genes (*GAPDH* and *Myosin*) were employed. The reactions were repeated three times, and the means were used to calculate the expression.

### Subcellular localization of the six candidate genes

The coding sequences of the candidate genes were cloned into *pCAMBIA2300-35S-eGFP* vector. The constructs were respectively transformed into *N. benthamiana* leaves respectively. After 36-48h of infiltration, samples were examined by a confocal laser scanning microscope (LSM800, Carl Zeiss) for GFP signal detection.

## Results and analysis

### Weighted co-expression network screened 26 gene clusters with the same expression trend under nutrition and disease treatments

We collected all accessible transcriptome data from maize, rice, wheat and Arabidopsis thaliana root and leaf tissues under LN, LP and disease treatments till 2021, respectively, for the construction of weighted co-expression networks (**Supplementary Table 4** and **Supplementary Table 5**). Because of the considerable collinear link between maize and wheat, rice as well as Arabidopsis thaliana (**Supplementary Figure 1**), we performed a homology comparison of wheat, rice and Arabidopsis thaliana genes with maize genes to obtain the maize homologous genes and constructed the TPM matrix. A total of 105 samples of root tissue treated with LN, LP or diseases were used for analysis (**Supplementary Figure 2A**). After filtering out the genes with a deletion rate of more than 40%, 18538 genes were extracted from the root for analysis. The co-expression networks yielded a total of 22 modules (**Supplementary Figure 3A**). The number of genes in the 22 modules spans from 35 to 4904, with the black module having the most (4904 genes) and the brown2 module having the fewest (35 genes). A clear correlation relationship was found between the expression of genes in the modules (**Supplementary Figure 3B**). Similarly, 127 leaf tissue samples from N, P, and disease stress treatments met the requirements (**Supplementary Figure 2B**). WGCNA yielded four modules with a total of 18832 genes in the leaves (**Supplementary Figure 4**). Blue modules contain the most genes (10323), whereas red modules include the fewest (159).

**Figure 1:**
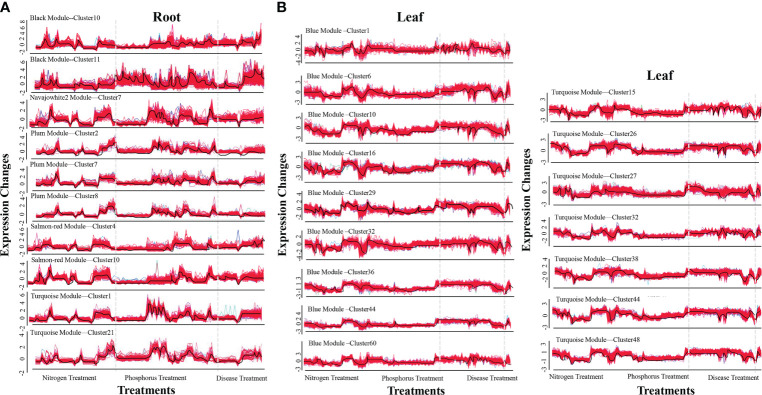
The most effective clusters of co-expression modules in roots and leaves. **(A)** Optimal root clusters. **(B)** Optimal leaf clusters. The ordinate represents the fold changes in expression. The abscissa represents different processing treatments. The black curves in the figure reflect a change in trend.

The R-packet MFUZZ was used to cluster the genes in each module in order to make a more systematic examination of the genes in the same module. The root results are as follows: (**Figure 1A**). First, two optimal clusters were obtained in the black module (c=20), namely cluster 10 (112 genes) and cluster 11 (196 genes). Cluster 7 was the only optimum cluster in the navajowhite2 module (c=10) (111 genes). In the plum module (c=10), three optimal clusters were screened: cluster 2 (94 genes), cluster 7 (96 genes), and cluster 8 (93 genes). Cluster 4 (101 genes) and 10 (105 genes) were the two optimal clusters in the salmon red module (c=20). In the turquoise module (c=26), Cluster 1 (120 genes) and cluster 21 (46 genes) were the two optimal clusters. The blue module in leaf yielded nine candidate clusters: cluster 1 (116 genes), cluster 6 (230 genes), cluster 10 (100 genes), cluster 16 (352 genes), cluster 29 (214 genes), cluster 32 (200 genes), cluster 36 (359 genes), cluster 44 (389 genes), and cluster 60 (164 genes) (**Figure 1B**). Seven potential clusters were produced in the turquoise module (c=48), including cluster 15 (198 genes), cluster 26 (439 genes), cluster 27 (158 genes), cluster 32 (281 genes), cluster 38 (238 genes), cluster 44 (370 genes), and cluster 48 (470 genes) (**Figure 1B**). WGCNA and MFUZZ results successfully clarified the expression rules of a large number of genes in hundreds of samples, revealing that many genes have the law of simultaneous up-regulation or down-regulation under nutritional and disease stress.

### Meta-analysis screened out 22 scQTLs associated with nutrition and disease in maize

**Figure 2:**
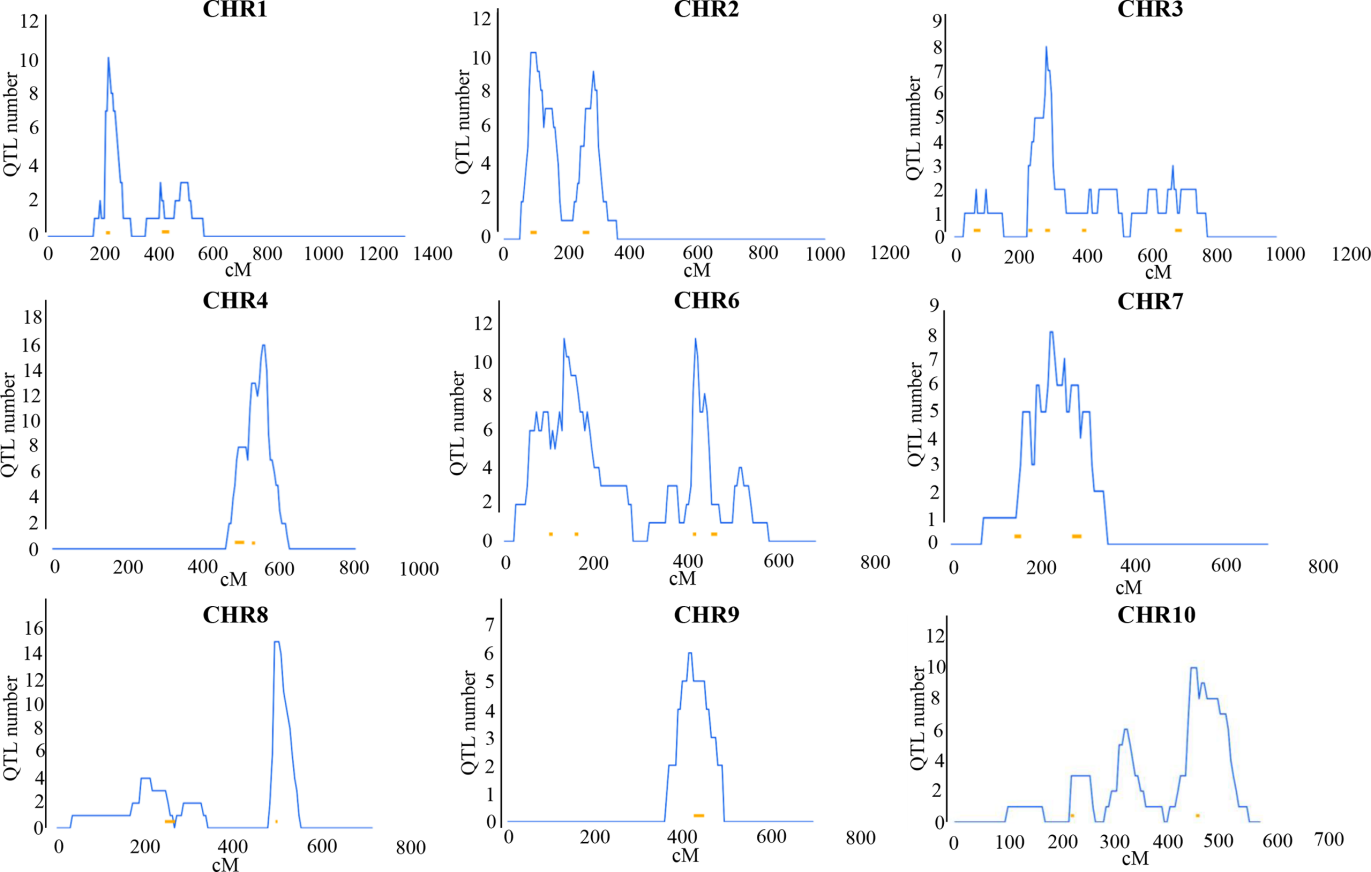
Distribution of 22 scQTLs on reference map IBM2 2008 neighbors. Vertical coordinates represent QTL numbers and horizontal coordinates reflect linkage distances of the reference map. The curves on these maps represent the density of QTLs on each chromosome. The lengths and placements of bold lines are subjective and should only for rough reference only.

This study included all available QTL information in maize under LN, LP and disease treatments (**Supplementary Table 6**). The collected QTL loci were integrated, yielding 49 nutrition and disease-related cQTLs. However, because these cQTLs may control the traits themselves, we also collected data on QTL loci detected under normal conditions (**Supplementary Table 7**). 51 cQTLs were eliminated using the same integrated strategy. If the cQTLs under treatments and normal conditions overlap, this nutrition and disease-related cQTL locus will be deleted. Finally, 22 cQTL loci were identified which are specifically related to nutrition and disease (scQTL) (**Figure 2** and **Table 1**).

**Table 1:** The information of the 22 scQTLs.

No	Name^a^	Bin	Left maker	Right maker	cQTL region (cM)	Physical region (bp)	Number of initial QTL
1	scQTL_1ND	1.03	aic3	IDP4348	210.8-215.85	33,322,465- 34,678,054	14
2	scQTL_1-1ND	1.05	bnlg1884	TIDP7049	414.9-423.99	91,511,981- 94,884,787	5
3	scQTL_2ND	2.02	pks1	IDP54	94-109.6	8,724,139- 10,021,379	11
4	scQTL_2-1ND	2.04	wt1	gams1	259.81-270.4	32,452,526- 34,353,587	12
5	scQTL_3ND	3.02	ereb103	gpm854	66-90.34	4,660,995- 6,945,972	3
6	scQTL_3-1ND	3.04	umc2584a	tis903.6a	282.55-290.37	119,999,294- 123,288,759	9
7	scQTL_3-2ND	3.05	pza00828	umc2265	347.25-354	158,938,555- 160,561,442	3
8	scQTL_3-3ND	3.06	AI770873	cl32876_1	388.1-391.88	167,726,479- 169,123,896	3
9	scQTL_3-4ND	3.08	a3	cah2	674.88-688.4	214,980,803-215,518,935	6
10	scQTL_4ND	4.08	gpm727c	rz596b	534.3-539	195,089,318-195,374,901	13
11	scQTL_4-1ND	4.08	npi570	IDP276	480.7-506	185,890,136-187,024,847	5
12	scQTL_6ND	6.01	pmei14	IDP87	95.6-100.27	57,286,137-38,086,440	12
13	scQTL_6-1ND	6.03	arid8	bnl (tas1i)	162.8-168.66	98,248,764-103,293,200	15
14	scQTL_6-2ND	6.06	gras62	npi102	409.2-413.1	156,946,311-157,609,193	15
15	scQTL_6-3ND	6.07	bcd828b (atpb)	IDP8054	460.55-470.87	165,352,073-162,860,116	4
16	scQTL_7ND	7.02	mmc0162	gpm836	156.28-165.15	19,761,619-21,811,964	11
17	scQTL_7-1ND	7.02	IDP1424	bnlg657	271.19-284.13	126,840,182-127,184,226	9
18	scQTL_8ND	8.03	umc1984	gpm456b	234.8-250.11	79,810,748-92,690,815	9
19	scQTL_8-1ND	8.07	IDP1495	AY110539	492.85-494.2	168,374,785-168,605,586	17
20	scQTL_9ND	9.06	bnlg1091	csu28a (rpS22)	415.05-436	140,610,188-142,755,422	7
21	scQTL_10ND	10.03	csu213b	umc155	210.09-226.1	75,822,918-87,175,007	5
22	scQTL_10-1ND	10.05	gpm712d	TIDP3023	451.13-458.53	143,987,393-147,334,795	13

a The names of scQTL are based on their respective chromosomes and the processing treatments. For example, scQTL_1ND is located on chromosome 1, the number after the underline indicates the order of this cQTL, and ND stands for nutrition and disease treatments.

### Candidate genes in co-expression modules and scQTLs are enriched in multiple stress-resistant related pathways

The investigation of genes in clusters and scQTLs using GO and KEGG PATHWAY revealed that these genes were primarily involved in the rRNA metabolic process, RNA binding, DNA metabolic process, nucleic acid metabolism, and cellular aromatic compound metabolism (**Supplementary Figure 5** and **Supplementary Table 8**). The majority of the concrete metabolic pathways are potentially involved in stress resistance. It mainly includes MAPK signaling pathway, phenylpropanoid biosynthesis, plant-pathogen interaction, diterpenoid biosynthesis, terpenoid backbone biosynthesis, ubiquinone and another terpenoid-quinone biosynthesis, pentose phosphate pathway, and nitrogen metabolism (**Supplementary Figure 6**). Most of these pathways are involved in stress-resistant signals. The results enhanced the reliability that a particular number of genes in co-expression modules and scQTL loci respond to P, N and disease stress.

### The consistent genes in clusters and scQTL loci

Among the 195 common candidate genes in the optimum clusters and scQTL loci (**Supplementary Table 9**), six candidate genes (Zm00001d038432, Zm00001d047747, Zm00001d009941, Zm00001d009866, Zm00001d009824, and Zm00001d00990) simultaneously present in the optimal clusters of roots and leaves and scQTL loci were screened out. Moreover, the six genes were also found to be closely related to GLS and CLB in scQTLs (**Table 2**). Because GLS is a high incidence disease in the southwest hilly corn region of China, we took these six genes as important candidate genes for further experimental verification (**Table 2**), and preliminarily investigated the relationship between LN, LP treatments and GLS treatments.

**Table 2:** Six genes were simultaneously screened in root and leaf clusters and scQTLs.

Gene name	Root module	Root cluster	Leaf module	Leaf cluster	scQTL^a^	Traits
Zm00001d047747	navajowhite2	cluster7	blue	cluster16	scQTL_9ND	**GLS,** DS, DA
Zm00001d038432	black	cluster4	blue	cluster36	scQTL_6-1ND	**GLS**, ELA, MRL, GNPE, KN, GY, et al
Zm00001d009941	navajowhite2	cluster7	blue	cluster36	scQTL_8ND	**GLS**, ASI, ELL, JB, SB, CLB
Zm00001d009866	navajowhite2	cluster7	blue	cluster44	scQTL_8ND	
Zm00001d009824	plum	cluster7	blue	cluster44	scQTL_8ND	
Zm00001d009903	plum	cluster8	blue	cluster44	scQTL_8ND	

a The names of scQTL are based on their respective chromosomes and the processing treatments. For example, scQTL_1ND is located on chromosome 1, the number after the underline indicates the order of this cQTL, and ND stands for nutrition and disease treatments. ASI, anthesis-silking interval; CLB, corn leaf blight; DA, days to anthesis; DS, days to silking; ELA, leaf area; ELL, ear leaf length; GLS, gray spot disease; GNPE, grain number per ear; GY, grant yield; JB, jointing stage biomass; KN, number of grains per row; MRL, max toot length; SB, seedling biomass.

### Expression pattern of these six candidate genes

Firstly, these six candidate genes were subcellular localized in *Nicotiana benthamiana*. The results showed that Zm00001d009866 and Zm00001d009824 are located in the endoplasmic reticulum and nucleus respectively. Zm00001d009903 was targeted to the membrane and mitochondria. Zm00001d047747 and Zm00001d038432 were identified in the membrane, whereas Zm00001d009941 was found to be expressed on mitochondria (**Supplementary Figure 7**).

**Figure 3:**
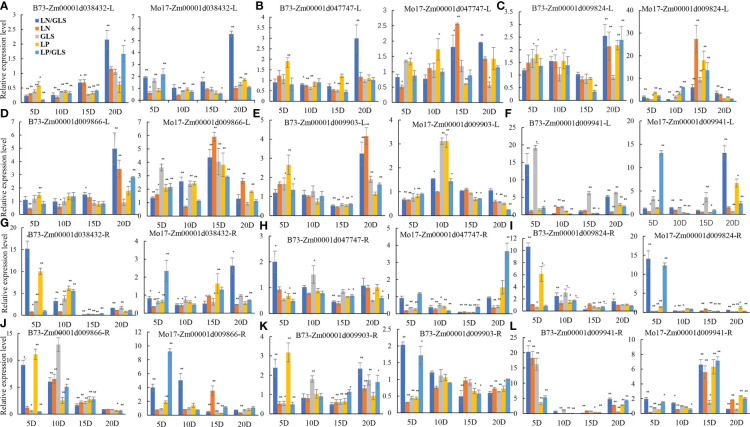
Expression patterns of six candidate genes in B73 and Mo17 under different treatments. **(A, G)** expression of Zm00001d038432 in root and leaf of B73 and Mo17. **(B, H)** expression of Zm00001d047747 in root and leaf of B73 and Mo17. **(C, I)** expression of Zm00001d009824 in root and leaf of B73 and Mo17. **(D, J)** expression of Zm00001d009866 in root and leaf of B73 and Mo17. **(E, K)** expression of Zm00001d009903 in root and leaf of B73 and Mo17. **(F, L)** expression of Zm00001d009941 in root and leaf of B73 and Mo17. R, Root; L, Leaf; GLS, Gray spot treatment; LN, Low nitrogen treatment; LP, Low phosphorus treatment; LN/GLS, Simultaneous treatment of low nitrogen and gray spot disease; LP/GLS, Simultaneous treatment of low phosphorus and gray spot disease. ** Expression that is exceedingly significant; * expression that is significant.

Real-time quantitative PCR was used to confirm the response of the six candidate genes to LN, LP, and GLS stress (**Figure 3**). The gene expression of Zm00001d038432, Zm00001d047747, Zm00001d009824, Zm00001d009866 and Zm00001d009903 in the leaves increased significantly in the late stage of stress treatments in B73 and Mo17 materials (**Figures 3A–E**). Zm00001d009941 expression was found to be 15-fold higher in B73 roots and leaves after LN/GLS and GLS treatments (**Figures 3F–L**). Both in the early stage of LP/GLS treatments and in the later stage of LN/GLS treatments, the expression in Mo17 leaves was dramatically up-regulated by more than tenfold (**Figures 3F, L**). The expression levels of these six potential genes changed more dramatically in the root system than in the leaves (**Figures 3G–L**). Interestingly, a consistent pattern of candidate genes mostly responding to stress at the early and middle phases was discovered (**Figures 3G–L**), implying the rapid response mechanism of the root system to nutrition stress and the crisscross of signaling between nutrients and diseases.

### The candidate genes exhibit synergistic expression under nutrition and GLS treatments

**Figure 4:**
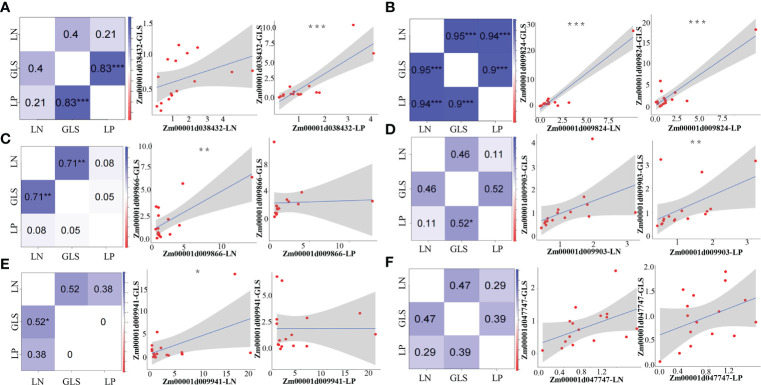
Pearson correlation analysis between nutrition and GLS treatments of candidate genes. **(A)** Correlation results of Zm00001d038432 under nutrition and GLS treatments. **(B)** Correlation results of Zm00001d009824 under nutrition and GLS treatments. **(C)** Correlation results of Zm00001d009866 under nutrition and GLS treatments. **(D)** Correlation results of Zm00001d009903 under nutrition and GLS treatments. **(E)** Correlation results of Zm00001d009941 under nutrition and GLS treatments. **(F)** Correlation results of Zm00001d047747 under nutrition and GLS treatments. LP, Low phosphorus; LN, Low nitrogen; GLS, Gray leaf spot. *5% significance level, **1% significance level, ***0.1% significance level.

We ran a correlation coefficient analysis on the expression levels of each candidate gene under the LP and GLS treatments or the LN and GLS treatments to confirm the synergistic link of the candidate genes between nutrition and GLS stress. Both genes Zm00001d038432 and Zm00001d009903 had a very significant positive correlation between LP and GLS treatments, while no correlation was detected between LN and GLS treatments (**Figures 4A, D**). The gene Zm00001d009824 showed a highly significant positive correlation between LP and GLS, LN and GLS, as well as LP and LN treatments (**Figure 4B**). Both the Zm00001d009866 and Zm00001d009941 genes exhibited a very significant positive correlation between LN and GLS treatments (**Figures 4C, E**). However, no synergistic expression of Zm00001d047747 was found between nutrition and disease (**Figure 4F**), which could be attributed to the too few processing time nodes selected in this study.

## Discussion

Previously, we found that the LP can induce changes in secondary metabolites related to plant immunity using multi-omics (Luo et al., 2019), which is consistent with another study that reported a close relationship between the activity of the central regulator PHR1 in response to low phosphorus stress and plant immune system (Castrillo et al., 2017). To explore candidate genes that are closely related to nutrition and disease immunity on a genome-wide scale, we collected all relevant transcriptome and QTL mapping data, and then integrated the two results, namely the results of reverse and forward genetics, to screen out the candidate genes with the synergistic expression relationship under nutrition and disease treatments. WGCNA has been proved to be an efficient data mining method since it can selectively screen out candidate genes associated with target traits and perform modular classification to get co-expression modules with high biological significance (Mishra et al., 2021). Meta-QTL analysis can integrate QTL information from different experiments and groups, narrow the confidence interval, and improve the accuracy of QTL mapping (Goffinet and Gerber, 2000). Therefore, combining them can greatly improve the effectiveness of candidate gene mining for nutrition and diseases that are synergistically associated.

### Nutrition can influence disease-related phenotypic changes

We investigated the characteristics of phenotypic alteration in both B73 and Mo17 at 15 and 20 days following nutritional and disease treatments, respectively. The results showed that the lesion area of gray spot was bigger under LN treatments than under normal nutrition conditions (**Supplementary Figures 8A–D**).Clear yellowish leaves and purplish stems were observed under the LN treatments. The two materials were withered and died at the inoculation site of leaves after the 20-day LN treatment, and the purple hue on the stem deepened, but the area of lesions did not expand (**Supplementary Figures 8C, D**). Both B73 and Mo17 materials had reduced lesions area under the LP treatments than those under the normal nutrition treatments (**Supplementary Figures 8E–H**).The leaves turned pale yellow after 15 days of LP treatments, and the stems turned lavender as well (**Supplementary Figures 8E, F**). In day 20 of treatments, a few yellow leaves developed on the lower part of the plant, the purple color of the stem became slightly darker than on day 15, and the size of the leaf lesions remained relatively constant (**Supplementary Figures 8G, H**). The findings indicated that nitrogen-deficient plants are weaker, develop slower, age faster, and are more susceptible to pathogens (Snoeijers et al., 2000). However, no apparent expansion of lesions was observed for the incidence of gray spot disease under LP which is consistent with earlier studies on rice that increasing phosphorus fertilizer diminishes rice’s ability to resist black spot disease (Sharma and Kolte, 1994). More research is needed to verify the mechanism of action of LP on GLS disease.

### Functional prediction of the synergistic mechanism between nutrition and disease of these candidate genes

According to the relevant literatures, the six genes and their homologous genes screened in this study have not been functionally validated. Therefore, we predicted the biological functions of each gene’s promoter and structural domains. The candidate genes’ promoter elements analysis revealed that they contain cis-acting elements such as cis-acting regulatory element involved in the MeJA-responsiveness, cis-acting element involved in defense and stress responsiveness (Yu et al., 2018), and so on. It is speculated that the candidate genes may have potential functions in stress resistance. Furthermore, a functional analysis of gene domains revealed that Zm00001d038432 is primarily composed of Pkinase (protein kinase domain) and LRRNT_2 (leucine-rich repeat sequence). Protein kinase primarily introduces phosphate groups into proteins and functions as a universally important coenzyme and enzyme regulator to bind with ATP and adrenal Glycosides bind 5’-triphosphates. Signal transduction, cell adhesion, DNA repair, recombination, transcription, RNA processing, disease resistance, apoptosis, and immune responses are all mediated by proteins containing LRRs (Rothberg et al., 1990; Roux et al., 2011; Araya et al., 2014). Because these genes are all new and unstudied, the information available for reference is limited, and their functions must be identified by subsequent genetic methods of transgenes.

## Conclusions

Investigating the signal cross between nutrition and disease may potentially aid in disease control in the field by coordinating fertilizer use. We screened six synergistic response genes based on WGCNA and cQTLs results under nutrition and disease treatment conditions in this investigation. The current findings, however, are insufficient to explain the functions and regulatory processes of these six candidate genes in synergetic control of nutrition and disease related traits. Transgenic techniques and other molecular biological methods will be used in the future to further our understanding of them.

## Data availability statement

The datasets presented in this study can be found in online repositories. The names of the repository/repositories and accession number(s) can be found in the article/**Supplementary Material**.

## Author contributions

BwL and SbG conceived and designed the experiments. BwL and JL wrote the manuscript and prepared the figures and tables. JL and ByL contributed to data collection and bioinformatics analysis. All the authors performed the experimental verification. BwL, JL, HZ, and SbG reviewed drafts of the paper. All authors contributed to the article and approved the submitted version.

## Funding

This research was supported by the Project Funded by National Key Research and Development Program of China (grant no. 2021YFF1000500 and 2021YFD1200704), the Natural Science Foundation of China (grant no. 32101655), Sichuan Science and Technology Support Project (grant no. 2021YFYZ0027, 2021YFYZ0020), and also supported by the earmarked fund for China Agriculture Research System (grant no. CARS-02-09).

## Acknowledgments

We thank Dr. Yuzhou Lan (The Swedish University of Agricultural Sciences, Lomma, Sweden) and Dr. Peng Ma (Sichuan Academy of Agricultural Sciences, Mianyang, China) for kindly providing advice on article writing and language revision. We also thank Drs. Xiangling Lv (Shenyang Agricultural University, Shenyang, China) for providing *Cercospora zeae-maydis*.

## Conflict of interest

The authors declare that the research was conducted in the absence of any commercial or financial relationships that could be construed as a potential conflict of interest.

## Publisher’s note

All claims expressed in this article are solely those of the authors and do not necessarily represent those of their affiliated organizations, or those of the publisher, the editors and the reviewers. Any product that may be evaluated in this article, or claim that may be made by its manufacturer, is not guaranteed or endorsed by the publisher.
