# Supplementary material for: Mining synergistic genes for nutrient utilization and disease resistance in maize based on co-expression network and consensus QTLs
Source: Front Plant Sci. 2022 Oct 28;13:1013598. doi: 10.3389/fpls.2022.1013598 (PMC9650340; doi:10.3389/fpls.2022.1013598)
Supplement: Supplementary file 2 [file DataSheet_2.pdf]

Supplementary Material

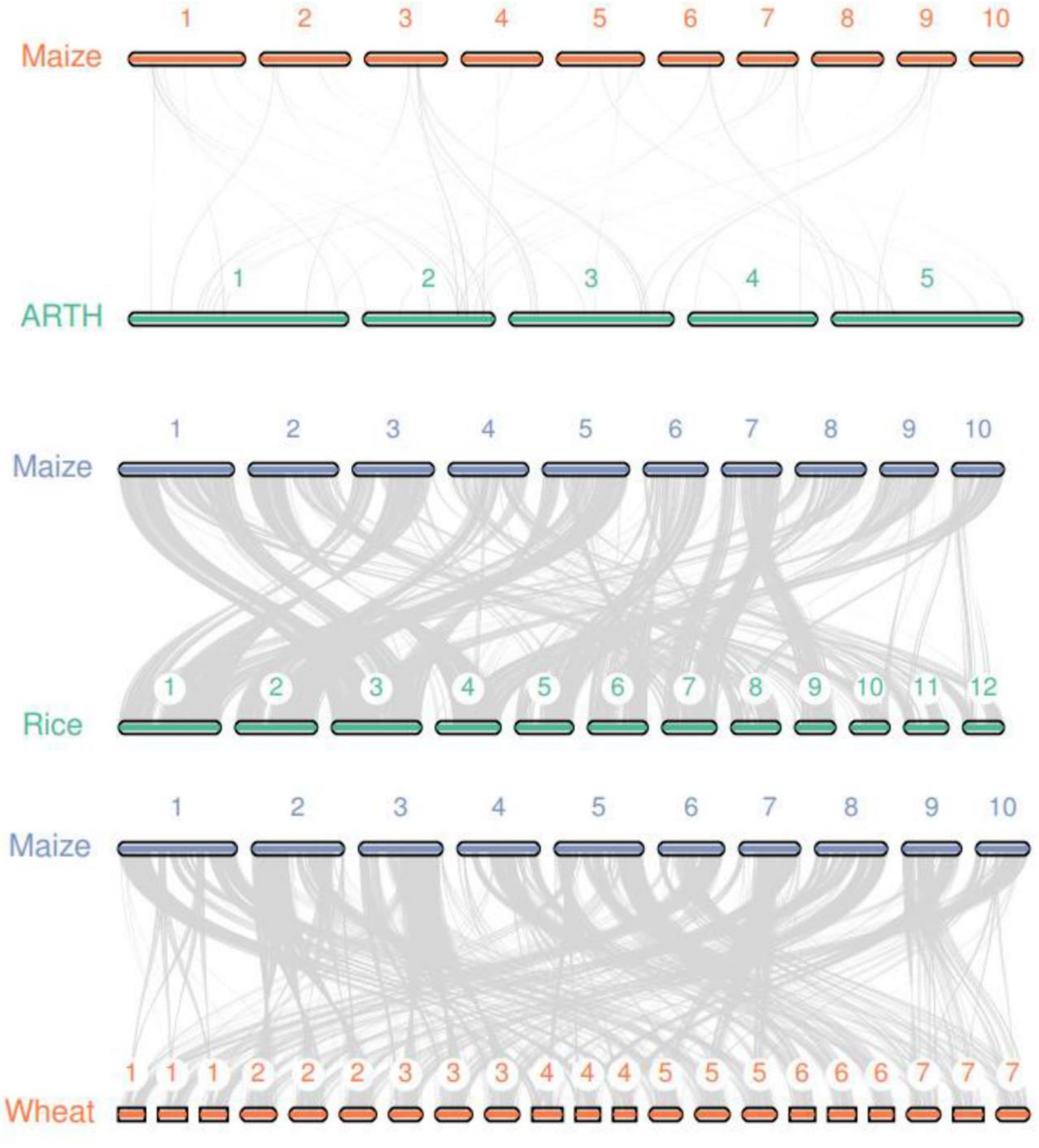

Supplementary Figure 1. Collinear relationship between maize and Arabidopsis, rice and wheat. ARTH: Arabidopsis.

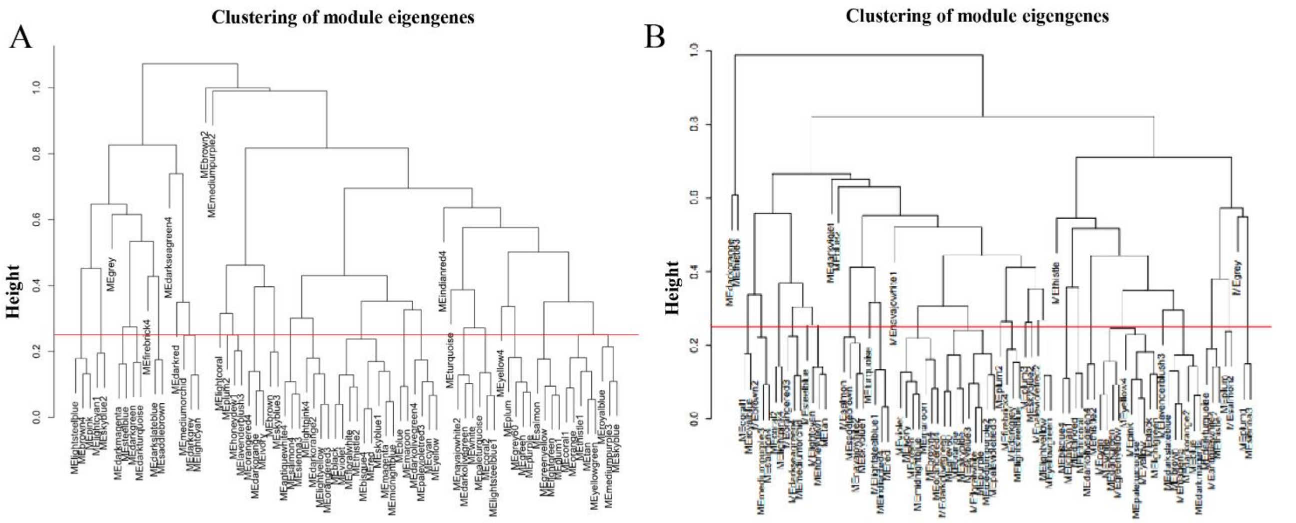

Supplementary Figure 2. Clustering dendrogram of samples. (A) Sample clustering of root. (B) Sample clustering of leaf. The ordinate represents the cluster height of genes.

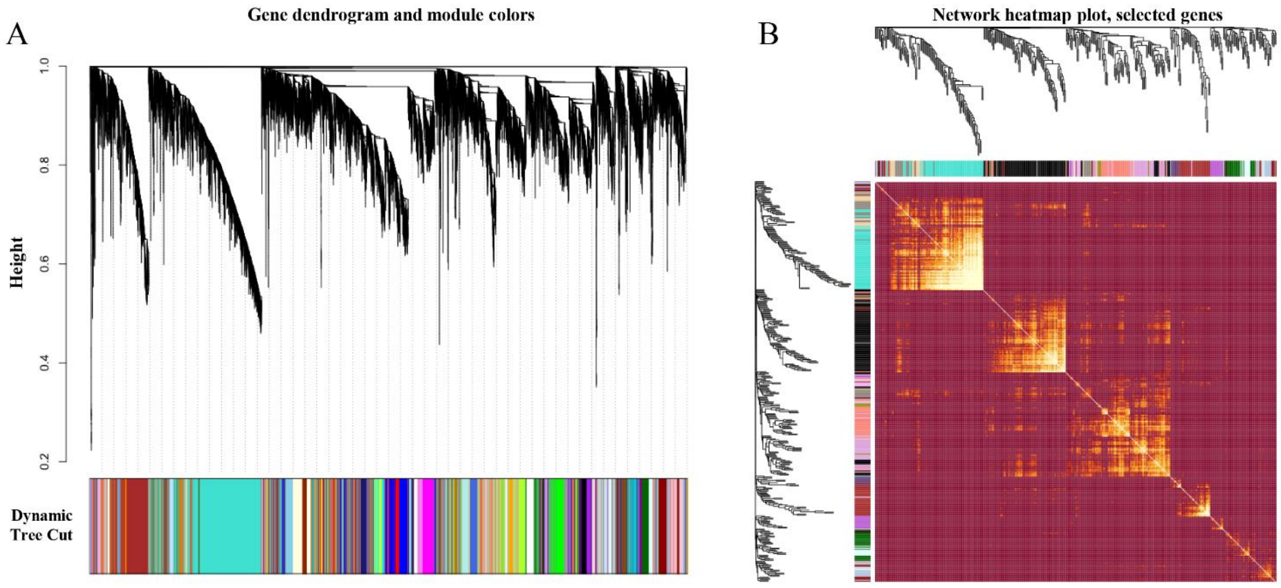

Supplementary Figure 3. Gene cluster dendrograms and module detecting of root tissues. (A) Cluster diagram of identified modules. (B) Heat map of identified modules.

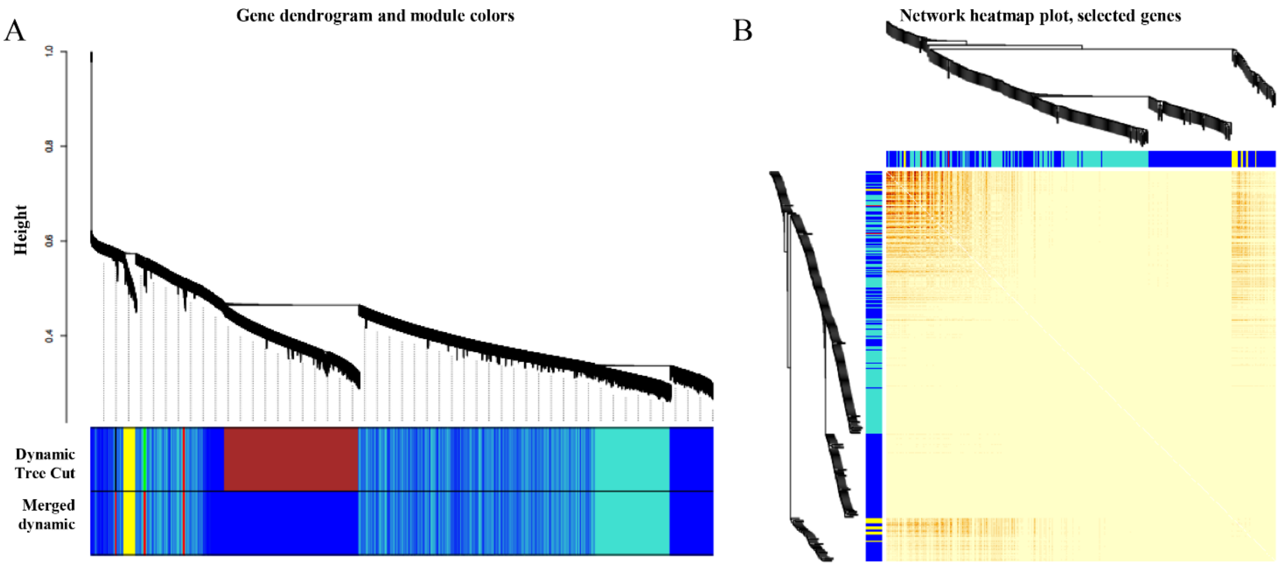

Supplementary Figure 4. Gene cluster dendrograms and module detecting of leaf tissues. (A) Cluster diagram of identified modules. (B) Heat map of identified modules.

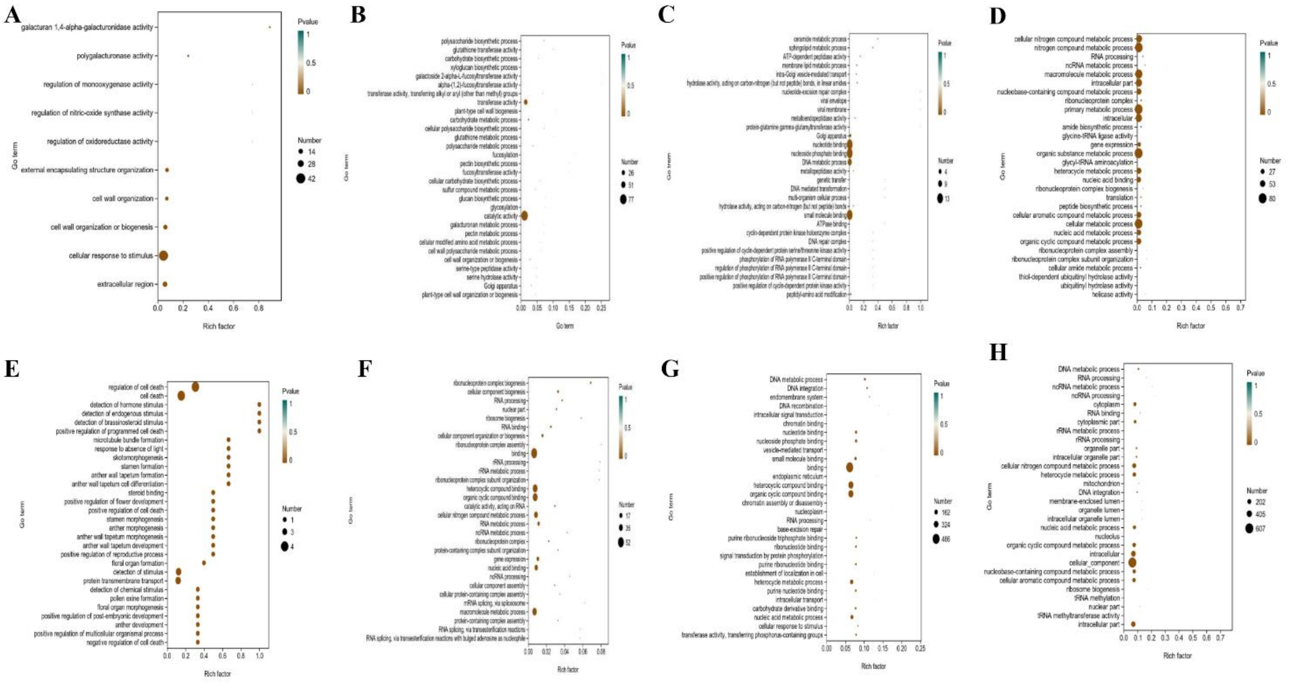

Supplementary Figure 5. GO analysis of genes in candidate Co-expression modules and scQTLs. (A) genes in scQTLs. (B) genes in root black co-expression module. (C) genes in root navajowhite2 co-expression module. (D) genes in root plug co-expression module. (E) genes in root salmon co-expression module. (F) genes in root turquiose co-expression module. (G) genes in leaf blue co-expression module. (H) genes in leaf tuquiose co-expression module.

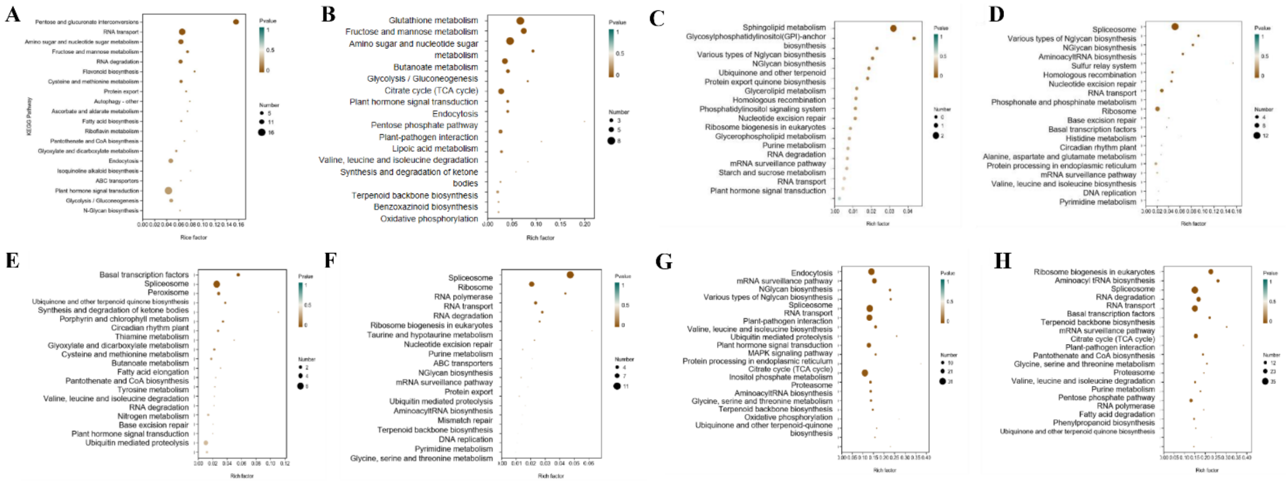

Supplementary Figure 6. KEGG analysis of genes in candidate Co-expression modules and scQTLs. (A) genes in scQTLs. (B) genes in root black co-expression module. (C) genes in root navajowhite2 co-expression module. (D) genes in root plug co-expression module. (E) genes in root salmon co-expression module. (F) genes in root turquiose co-expression module. (G) genes in leaf blue co-expression module. (H) genes in leaf tuquiose co-expression module.

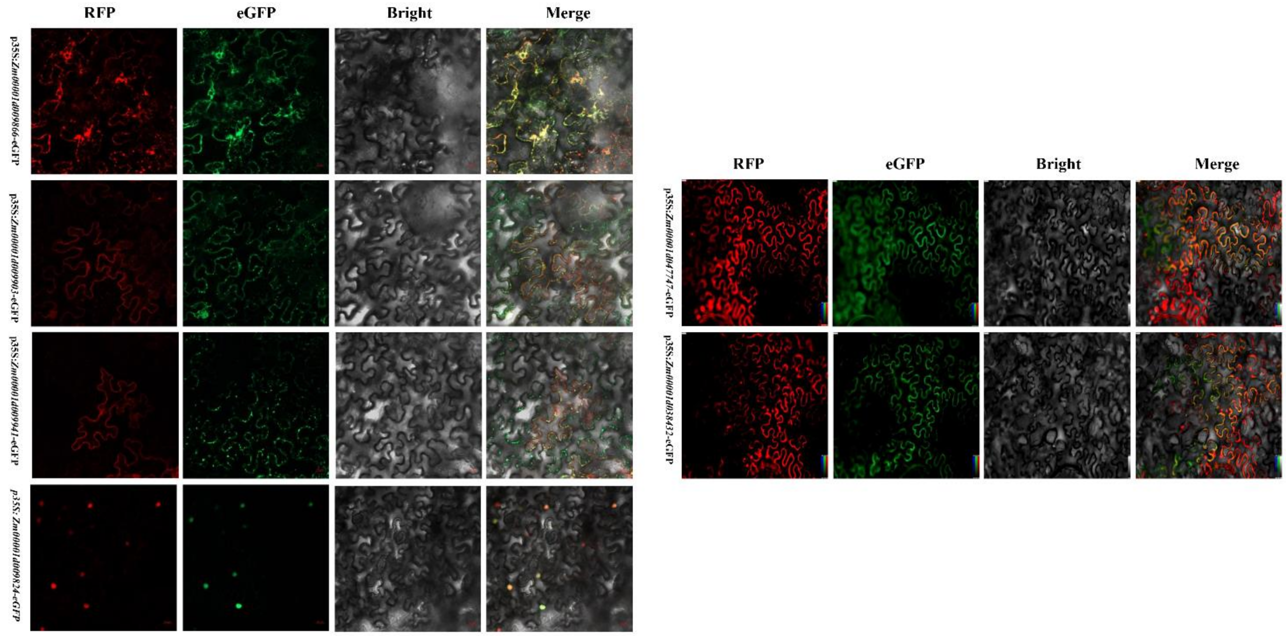

Supplementary Figure 7. Subcellular localization of candidate genes.

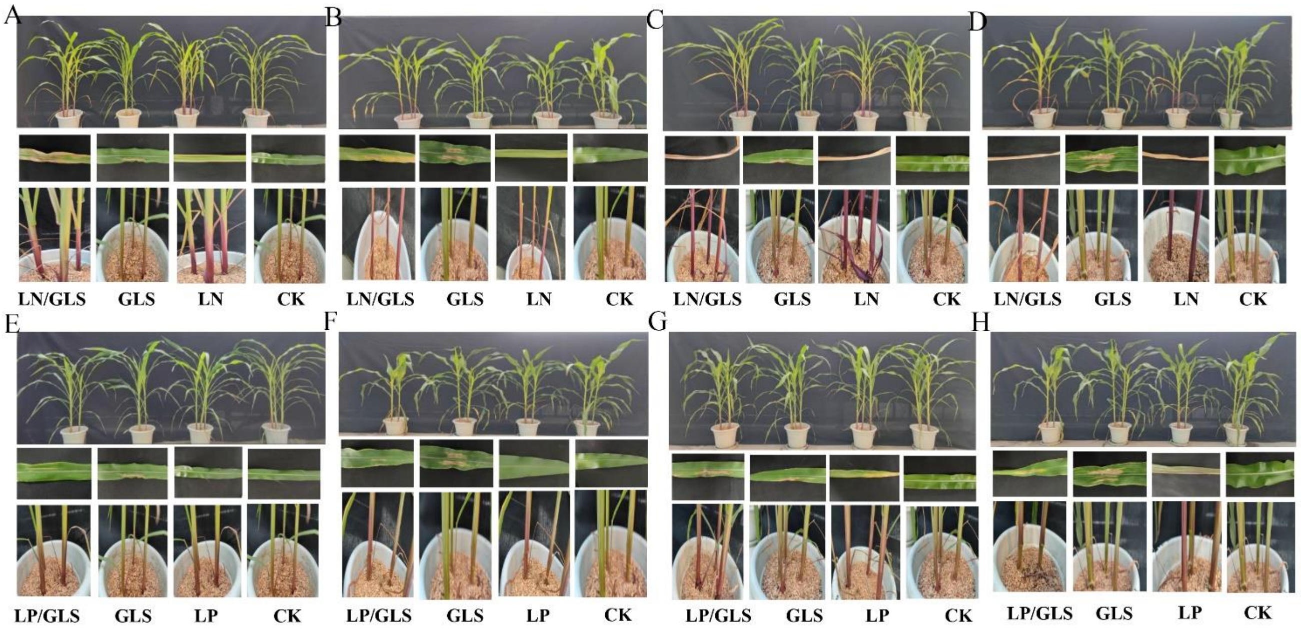

Supplementary Figure 8. Phenotypic results of B73 and Mo17 with different treatments. (A) B73, low nitrogen and gray spot treatment for 15 days. (B) Mo17, low nitrogen and gray spot treatment for 15 days. (C) B73, low nitrogen and gray spot treatment for 20 days. (D) Mo17, low nitrogen and gray spot treatment for 20 days. (E) B73, low phosphorus and gray spot treatment for 15 days. (F) Mo17, low phosphorus and gray spot treatment for 15 days. (G) B73, low phosphorus and gray spot treatment for 20 days. (H) Mo17, low phosphorus and gray spot treatment for 20 days.
